# Nitrogen starvation induces distinct photosynthetic responses and recovery dynamics in diatoms and prasinophytes

**DOI:** 10.1371/journal.pone.0195705

**Published:** 2018-04-11

**Authors:** Justin D. Liefer, Aneri Garg, Douglas A. Campbell, Andrew J. Irwin, Zoe V. Finkel

**Affiliations:** 1 Department of Geography and Environment, Mount Allison University, Sackville, New Brunswick, Canada; 2 Department of Biology, Mount Allison University, Sackville, New Brunswick, Canada; 3 Department of Mathematics and Computer Science, Mount Allison University, Sackville, New Brunswick, Canada; Stazione Zoologica Anton Dohrn, ITALY

## Abstract

Nitrogen stress is an important control on the growth of phytoplankton and varying responses to this common condition among taxa may affect their relative success within phytoplankton communities. We analyzed photosynthetic responses to nitrogen (N) stress in two classes of phytoplankton that often dominate their respective size ranges, diatoms and prasinophytes, selecting species of distinct niches within each class. Changes in photosynthetic structures appeared similar within each class during N stress, but photophysiological and growth responses were more species- or niche-specific. In the coastal diatom *Thalassiosira pseudonana* and the oceanic diatom *T*. *weissflogii*, N starvation induced large declines in photosynthetic pigments and Photosystem II (PSII) quantity and activity as well as increases in the effective absorption cross-section of PSII photochemistry (*σʹ*_PSII_). These diatoms also increased photoprotection through energy-dependent non-photochemical quenching (NPQ) during N starvation. Resupply of N in diatoms caused rapid recovery of growth and relaxation of NPQ, while recovery of PSII photochemistry was slower. In contrast, the prasinophytes *Micromonas* sp., an Arctic Ocean species, and *Ostreococcus tauri*, a temperate coastal eutrophile, showed little change in photosynthetic pigments and structures and a decline or no change, respectively, in *σʹ*_PSII_ with N starvation. Growth and PSII function recovered quickly in *Micromonas* sp. after resupply of N while *O*. *tauri* failed to recover N-replete levels of electron transfer from PSII and growth, possibly due to their distinct photoprotective strategies. *O*. *tauri* induced energy-dependent NPQ for photoprotection that may suit its variable and nutrient-rich habitat. *Micromonas* sp. relies upon both energy-dependent NPQ and a sustained, energy-independent NPQ mechanism. A strategy in *Micromonas* sp. that permits photoprotection with little change in photosynthetic structures is consistent with its Arctic niche, where low temperatures and thus low biosynthetic rates create higher opportunity costs to rebuild photosynthetic structures.

## Introduction

Among photosynthetic organisms phytoplankton are especially subject to rapid variation in light and nutrient conditions. Sustained changes in light between maximum availability and darkness occur on time scales of minutes, in coastal environments, to days in the open ocean [1]. Sustained nutrient depletion can be imposed on similarly short time scales following dense blooms or vertical mixing through the ocean surface mixed layer [2,3]. Yet the effects of dynamic nutrient stress on photosynthetic response are much less studied than the responses to varying light intensity under nutrient replete conditions. In phytoplankton, insufficient availability of nutrients such as nitrogen (N) can cause excess light absorption relative to the biosynthetic sinks for this energy, potentially resulting in photo-oxidative damage to a cell [4]. Thus under N stress, phytoplankton cells must control light absorption or the photochemical utilization of light energy to mitigate the damage that may be caused by this excess irradiance. How photosynthetic responses to nutrient stress vary across phytoplankton taxa, cell sizes, and ecological niches is poorly understood, which presents a major challenge for effectively modelling phytoplankton dynamics [5–7].

Photosynthetic responses vary with the extent and degree of acclimation to nutrient stress, which can range from chronic steady-state nutrient limitation to an abrupt onset of nutrient starvation [4,8]. We will herein use the term “starvation” to denote the non-steady state condition of nutrient stress following unbalanced growth and the term “limitation” to indicate steady state nutrient stress, with “stress” being the inclusive term for all non-replete conditions [4,8]. Experiments imposing nutrient limitation mimic nearly constant environments, and thus they may fail to characterize responses to dynamic ocean conditions in which nutrients can be rapidly exhausted or resupplied [9]. The unbalanced state of nitrogen starvation in phytoplankton cells appears to produce more acute photosynthetic responses than steady-state nitrogen limitation. Several important photosynthetic parameters including the quantum yield of Photosystem II (PSII) photochemistry (*F*_v_/*F*_m_) and net carbon production normalized to light absorption can be maintained across a wide range of severity of steady-state N limitation [4,8,10]. In contrast, the onset of nitrogen starvation can cause a large induction of energy-dependent photoprotective mechanisms and large drops in *F*_v_*/F*_m_, total cellular pigment content and the cellular content of PSII reaction centers [11–15]. The more rapid decline in PSII reaction centers relative to their associated antennae in N-starved cells may dramatically increase both the light absorption cross-section and the effective photochemical cross-section (*σ*_PSII_) for the remaining PSII centers [11,16]. These photosynthetic responses are part of an extensive reallocation of macromolecular components and overall metabolic response to N starvation that rapidly affects other primary cell functions such as respiration and resource acquisition [17]. The decline in photosynthetic pigments during N starvation coincides with a reallocation of resources to proteins involved in N acquisition, representing a large shift in an organism’s proteome [14, 18–20]. The reduction in photosynthetic pigments and proteins during N starvation also requires a reallocation of their associated lipids that make up thylakoid membranes as well as a diversion of carbon metabolism to the accumulation of storage lipids and carbohydrates [21–23].

Dynamic nutrient conditions can be expected to have varying impacts on photosynthetic responses across the considerable range of phytoplankton cell sizes and taxa. For example, N starvation may favour larger compared to smaller organisms [24,25] as larger organisms have more nutrient storage capacity and slower growth rates that lower their biomass-normalized N demand [25]. Additionally, larger phytoplankton cells have lower pigment-normalized light absorption and increased self-shading due to the package effect [26,27]. This excess photosynthetic light harvesting capacity in larger cells may provide more flexibility to reallocate N contained in photosynthetic pigment and protein content during N stress, with decreases in self-shading partially offsetting the effect of declining pigment content. Smaller cells may suffer from N starvation more rapidly than larger cells [24,25], but recover more quickly when N is resupplied due to their faster metabolic rates [28] and higher N affinity due to their higher surface-to-volume ratios [29,30].

Though cell size is likely a major factor in determining photosynthetic responses to N stress, taxa with distinct evolutionary histories have other size-independent differences in photosynthetic strategies. More rapid repair potential or lower intrinsic susceptibility to photoinactivation of PSII may be key differentiating features underlying a species’ strategy to survive extensive N starvation [16]. The particular strategy of a species for reallocating cellular N between N acquisition and photosynthetic components may also be important to resisting and recovering from N starvation [14]. Photoprotective mechanisms, such as cyclic electron flow around Photosystem I (PSI) or non-photochemical quenching (NPQ) through a xanthophyll cycle are induced during nutrient stress, yet the effectiveness of these mechanisms may vary considerably even among species of similar sizes and phylogeny [31–33].

Here we investigate photosynthetic responses during N starvation and recovery among phytoplankton taxa to assess the potential role of these responses in shaping phytoplankton communities. We examine two classes that often dominate their respective size ranges and also have distinct evolutionary histories, photosynthetic structures, and photoprotective mechanisms: diatoms and prasinophytes. The particular species selected also represent both different cell sizes and ecological niches within these classes. Diatoms seem to possess more robust photoprotection through NPQ [34,35] and greater flexibility in photosynthetic pigment and protein content relative to prasinophytes and other taxonomic groups [36–38]. Diatoms also have lower intrinsic susceptibility to photoinactivation of PSII reaction centers compared to prasinophytes, which appear to be particularly susceptible to primary photodamage of PSII [27,38]. Despite these observations, how photosynthetic structure and function would respond to N starvation among such distinct species is not clear and this knowledge would greatly enhance the ability to predict their relative success in an ocean where N is often variable or scarce. We hypothesized that photosynthetic structures and pigments would decline more in the larger cells within each class and more so in diatoms than prasinophytes during N stress due to their greater steady-state light absorption capacity and package effect [26]. We also hypothesized that the ability to recover would progressively decline with continued N stress in the coastal species, which occupy more nutrient-rich niches, while oceanic species may be better adapted to recover from prolonged N starvation through their photoprotective or resource allocation strategies. Our findings indicate that phylogeny and cell size may be strong predictors of changes in photosynthetic structure during N-starvation. However, species within a class of more similar cell size yet from distinct niches can vary considerably in their photoprotective strategies and recovery from N starvation.

## Materials & methods

### Culture conditions and study species

Cultures of the coastal diatom *Thalassiosira pseudonana* (strain CCMP 1335), the open ocean diatom *Thalassiosira weissflogii* (strain CCMP 1010), and the Arctic prasinophyte *Micromonas* sp. (strain CCMP 2099) were all obtained from the National Center for Marine Algae and Microbiota (NCMA). Cultures of the prasinophyte *Ostreococcus tauri* (strain OTH95, RCC745, isolated from Thau Lagoon, France) were obtained from the Roscoff Culture Collection (RCC). The respective sizes of each study species are provided in Table 1. All cultures were grown under a subsaturating irradiance of 85*μ*mol photons m^-2^ s^-1^ [39–42] at 18°C, except *Micromonas* sp., which was grown at 6°C. These temperatures were near optimum for growth in each strain. Light was provided by cool white fluorescent bulbs on a 12:12 day:night cycle. All species were grown in natural seawater (Cape Tormentine, Canada) with a salinity of ~32 ppt that was amended with a modified version of f/2 nutrient concentrations and then filter-sterilized (Pall Acropak 0.8/0.2 *μ*m capsule filter). The modified media contained half the f/2 concentrations of sodium phosphate, sodium silicate (not added to media for prasinophytes), f/2 trace metals solution, f/2 vitamins mix and 2 mM sodium bicarbonate. Media for the diatoms and media for prasinophytes contained 60 *μ*M and 120 *μ*M sodium nitrate, respectively. The diatoms were maintained at a lower biomass, and hence grown with less nitrate, as lower densities were necessary to maintain a pH below 9 at all growth phases. Both media types were adjusted to a pH of 7.95–8.00 with HCl prior to inoculation. All species were grown in triplicate 5L culture bottles (Pyrex) with stirring by PTFE stir bars at ~60 RPM and continuous bubbling with filter-sterilized (VWR, 0.2 *μ*m PES syringe filters) air. All species were maintained as optically-thin, semi-continuous batch cultures (dilution every 2–3 days) at nutrient-replete conditions and were considered to be in acclimated, balanced exponential growth at these conditions after a minimum of 10 generations with less than 15% variation in growth rate.

**Table 1:** Description of study species.

Species (Strain)	Phylum	Class	Cell Size (*μ*m^3^)	Habitat
***Thalassiosira pseudonana* (CCMP 1335)**	Ochrophyta	Bacillariophyceae	158 ± 23	Temperate coastal estuary
***Thalassiosira weissflogii* (CCMP 1010)**	Ochrophyta	Bacillariophyceae	1630 ± 215	Temperate open ocean
***Ostreococcus tauri* (OTH 95)**	Chlorophyta	Mamiellophycaeae	0.5 ± 0.2	Temperate coastal lagoon
***Micromonas* sp. (CCMP 2099)**	Chlorophyta	Mamiellophycaeae	1.8 ± 0.3	Polar continental shelf

Cultures were initially sampled at balanced, nutrient-replete exponential growth for cell composition, biooptics, and Chl *a* fluorescence analyses. Immediately after this sampling, cultures were diluted with N-free media (same media as described above without sodium nitrate added) to a predetermined cell density that would allow them to be at a similar optical density and pH in N-deplete stationary phases as observed during the initial exponential phase sampling. Cultures were sampled once again in the late exponential phase (twice in the case of *O*. *tauri*) when growth had declined, but not ceased. Sampling was conducted three more times in the stationary phase after growth had ceased, except in the case of *T*. *pseudonana* which had not ceased growth until the day after the first stationary phase sampling. The same parameters of cell composition, biooptics, and Chl *a* fluorescence analyses were determined for the late exponential and stationary phase samplings as with the initial exponential phase sampling.

### Recovery experiments

Growth experiments were initiated in parallel with each stationary phase sampling to assess recovery from N starvation in each species. At each stationary phase sampling, two 16ml aliquots of each triplicate culture were removed and diluted with 32 ml of fresh N-free media in a 50 ml culture tube. One aliquot from each triplicate culture received 5 *μ*l of concentrated sodium nitrate solution, creating a final concentration of 220 *μ*M nitrate, while the other received 5 *μ*l of deionized water and acted as a control. Recovery sub-cultures were maintained at the same light and temperature conditions as their respective batch cultures and cell density in each recovery experiment tube was measured daily. No detectable growth in all control cultures demonstrated that all stationary phase sub-cultures were N-starved and that growth in the treatment sub-cultures was due to the resupply of N.

### FRR and absorbance measurements

Fluorescence induction was measured by fast repetition rate fluorometry (FRRf) using a PSI FL-3500 fluorometer (Photon Systems Instruments, Drasov, Czech Republic) at growth temperature and induction curves were analyzed using the PSIWORX package for R (http://sourceforge.net). Fluorescence measurements consisted of 40, 1.2 *μ*s flashlets of blue light (455 nm, ~30,000–85,000 *μ*mol photons m^-2^ s^-1^) provide by an LED over 128 *μ*s to progressively close PSII reaction centers and induce maximum fluorescence. The relaxation of fluorescence and reopening of PSII reaction centers was then measured by the application of the same flashlet conditions every 100 *μ*s over a period of 0.4 s. Samples were then allowed 1 s to relax and the fluorescence induction-relaxation series was repeated. After initial measurement in the dark, an actinic light was applied for 10 s and the same fluorescence protocol was repeated, with this sequence being repeated at eight more increasing light levels.

All FRR fluorescence induction-relaxation data were analyzed with PSIWORX script (A. Barnett, http://source-forge.net/projects/psiworx) for R software to derive the following parameters: minimal fluorescence (*F*_*o*_), maximal fluorescence (*F*_m_), maximal fluorescence under actinic light equivalent to growth irradiance (*F*_m_’), minimal fluorescence at growth irradiance after 1s darkness (*F*_o_’_1s_), maximal fluorescence at growth irradiance after 1 s darkness (*F*_m_’_1s_), steady-state fluorescence at growth irradiance (*F*_s_), effective absorption cross-section of PSII (*σ*_PSII_), the coefficient of excitonic connectivity among PSII reaction centers (*ρ*), the slow lifetime of PSII reopening (*τ*_1_), and the fast lifetime of PSII reopening (*τ*_2_) [43–46]. The full definition and derivation of all photosynthetic parameters determined by FRRf are given in Table 2. These FRRf parameters were used to calculate the fraction of open PSII reaction centers (*q*_p_) [43], the rate of electron transfer from PSII (ETR) [47], and the quantity of active PSII reaction centers (PSII_Active_) [48]. To more accurately estimate minimal fluorescence at growth irradiance (*F*_*o*_’) used in calculations of *q*_p_, the increase from *F*_m_’ to *F*_m_’_1s_ was used to correct (*F*_o_’_1s_) according to the equation in Table 2 [44, 45]. The amplitude of initial fluorescence relaxation in each fluorescence induction-relaxation curve was used to combine *τ*_1_ and *τ*_2_ as a weighted sum indicating the overall lifetime of PSII reopening (*τ*). Measurements of (*F*_v_*/F*_m_), *σ*_PSII_, and *ρ* were compared under dark-acclimated and low actinic light conditions (8 and 21 *μ*mol photons m^-2^ s^-1^) to determine the best approximation of their maximum values across all growth phases. As a result, dark *F*_v_*/F*_m_ values are reported for all species and values of *σ*_PSII_ and *ρ* reported were determined at 21 *μ*mol photons m^-2^ s^-^1 for the diatom species and at 8 *μ*mol photons m^-2^ s^-1^ for the prasinophyte species (thus given as *σʹ*_PSII_ and *ρʹ*). However, the overall trends in each of these parameters across species and growth conditions were very similar under both dark-acclimated and low actinic light measurement conditions. Using FRRf parameters, we also computed a quantum yield for three possible fates of excitation energy [49,50] that entered the pigment-protein complexes of PSII: photochemistry by PSII (***Φ***_PSII_), dissipation as heat through regulated, energy-dependent NPQ mechanisms such as the xanthophyll cycle (***Φ***_NPQ_), and constitutive, energy-independent dissipation as heat and fluorescence (***Φ***_NO_) (Table 1).

**Table 2:** Parameters and definitions.

Parameter	Definition, Units	Derivation	Reference
***μ***_***max***_	Maximum growth rate under steady-state, N-replete conditions, days^-1^	Slope of ln(Nt)-vs.-time plot, where N_*t*_ is cell density at time *t*	** **
***T***_**R**_	Recovery time; estimated time until *μ*_max_ is restored following resupply of N to N-starved cultures	Time when *μ*_R_ = *μ*_max_ in a regression of *μ*_R_-vs.-time plot, where *μ*_R_ is recovery growth rate	** **
***RS***	Recovery competence score, dimensionless	(1/*T*_R_*)*/*μ*_max_	** **
***F***_**o**_	Minimum fluorescence yield, dark-acclimated state		[43]
***F***_**m**_	Maximum fluorescence yield, dark-acclimated state		[43]
***F***_**v**_	Variable fluorescence yield, dark-acclimated state	*F*m—*F*_o_	[43]
***F***_***s***_	Fluorescence under actinic light equal to growth irradiance	* *	[43]
***F***_**m**_***'***	Maximum fluorescence under actinic light equal to growth irradiance	* *	[43]
***F***_**m**_***'***_**1s**_	Maximum fluorescence 1s after excitation under actinic light equal to growth irradiance		[44,45]
***F***_**o**_***'***_**1s**_	Minimal fluorescence 1s after excitation under actinic light equal to growth irradiance		[44,45]
***F***_**o**_***'***	Estimated minimal fluorescence under actinic light equal to growth irradiance	*F*_o_'_1s_ * {1 - [(*F*_m_*'*_1s_ - *F'*_m_)/*F*_m_*'*_1s_]}	[44,45]
***F***_**v**_**/*F***_**m**_	Maximum potential quantum yield of PS II photochemistry	* *	[43]
***σ***_**PSII**_***'***	Effective absorption cross-section of PSII under low actinic light (Å^2^ quanta^-1^)	Exponential rate of rise in FRRf fluorescence induction curve	[46]
***σ***_**PSII**_***'***_**1s**_	Effective absorption cross-section of PSII under low actinic light (Å^2^ quanta^-1^) 1s after excitation under growth irradiance actinic light	Exponential rate of rise in FRRf fluorescence induction curve	
***ρ'***	Excitation connectivity between PSII reaction centers under low actinic light	Sigmoidicity of FRRf fluorescence induction curve	[46]
***q***_**p**_	Photochemical quenching of fluorescence, fraction of open PSII	(*F*_m_*'—F*_s_)/(*F*_m_'—*F*_o_')	[43]
**ETR**	Rate of electron transfer from PSII at growth irradiance (*I*), e^-^ PSII^-1^ s^-1^	*σ*_PSII_' * I * q_P_	[47]
**PSII**_**Active**_	Quantity of active PSII reaction centers at growth irradiance	*F*_o_*'*_1s_/ *σ*_PSII_*'*_1s_	[48]
**1/*τ***	Rate constant for reopening of PSII reaction centers, s^-1^	* *	[46]
***ϕ***_**PSII**_	Quantum yield of PSII photochemistry	(*F*_m_*'—F*_s_)/*F*_m_'	[49,50]
***ϕ***_**NO**_	Quantum yield of constitutive, energy-independent non-photochemical excitation energy dissipation	*F*_s_/F_m_	[49,50]
***ϕ***_**NPQ**_	Quantum yield of energy-dependent, regulated non-photochemical excitation energy dissipation	1 *-* (*ϕ*_PSII_ *+ ϕ*_NO_)	[49,50]

The absorbance spectrum for each species was measured at the same time as the FRRf measurements using the same dark-acclimated sample. Absorbance was measured from 400 – 700nm using a spectrophotometer (Olis Cary 14) utilizing a DSPC integrating cavity that provides near total internal reflectance over an effective pathlength of ~20cm. Absorbance measurements were corrected with a blank measurement made on the same sterile seawater media used for each culture.

Each species was evaluated prior to experiments to determine the dark-acclimation period (10, 15, 20, 30, or 45 minutes) that yielded the lowest value of the minimum fluorescence yield (*F*_0_) and the highest value of the maximum fluorescence yield (*F*_m_) in the dark-acclimated state. As a result, *T*. *pseudonana* and *T*. *weissflogii* were dark-acclimated for 30 minutes while *O*. *tauri* and *Micromonas* sp. were dark-acclimated for 20 minutes prior to fluorescence induction measurements. Additionally, *O*. *tauri* and *Micromonas* sp. were concentrated by low speed (<4000g) centrifugation at growth temperature prior to FRRf and absorbance measurements as they had an inherently low overall fluorescence yield. Aliquots of these cell concentrates were frozen immediately in liquid nitrogen for Chl *a* measurement. These additional Chl *a* measurements were used to normalize absorbance measurements. Chl *a* was extracted in a 3:2 mixture of 90% acetone:DMSO according to [51] and measured with a Turner AU-10 fluorometer [52] against a Chl *a* standard (Sigma). These fluorometric *in vitro* Chl *a* measurements were also made on unconcentrated cells of *O*. *tauri* and *Micromonas* sp. and all showed less than 5% difference from Chl *a* determined by HPLC as described above, thus all Chl *a* values or normalization reported have parity despite the use of two analytical methods.

### Particulate carbon and nitrogen, pigment, and nutrient analyses

Samples for quantification of particulate carbon and particulate nitrogen (CN) and pigments were filtered through Whatman GF/F filters (effective pore size, ~0.7 μm) under gentle vacuum pressure (<18 kPa or 5 in. Hg) and low light. Filters for CN analyses were pre-combusted (4 hours at 500°C). Samples for CN were placed on filter plates in a -20°C freezer immediately after collection and stored at this temperature. Frozen CN samples were dried at 60°C for 2 days, pelleted in pressed tin capsules and analyzed with a Costech CHN analyzer using acetanilide as a standard. Filtrate collected from pre-combusted GF/F filters during CN sample collection was analyzed for dissolved inorganic nitrogen (nitrate, nitrite, and ammonium) and phosphate colorimetrically using an autoanalyzer.

Filters collected for pigments were frozen immediately by immersion in liquid nitrogen and stored at -80°C until analysis. Pigment concentrations were measured by high performance liquid chromatography [53,54] on an Agilent 1100 HPLC (Agilent Technologies, Santa Clara, CA) against authenticated standards (DHI Lab, Horshølm, Denmark). The response factor for a lutein standard was used to quantify its epoxide precursor dihydrolutein [42,55]. For estimation of relative light harvesting antenna size, the pigments fucoxanthin and chlorophylls *c*_1_ and *c*_2_ (Chl *c*) were considered to be associated with fucoxanthin-chlorophyll protein complexes, the light harvesting antenna complexes of diatoms [56]. The pigments chlorophyll *b*, Mg-2,4-divinyl pheoporphyrin (MgDVP), prasinoxanthin, and dihydrolutein were considered to be associated with the light harvesting antenna complexes (Lhcp) complexes of both prasinophytes based on the Lhcp pigment stoichiometry of *Ostreococcus* [57] as both *Micromonas* and *Ostreococcus* are Clade II prasinophytes [58] with similar overall pigment profiles [55]. In diatoms, the de-epoxidation state of xanthophyll cycle pigments (DES) was calculated as DT/(DD + DT) where DT is diatoxanthin and DD is diadinoxanthin [34]. In prasinophytes, DES was calculated as [(0.5 * A) + Z]/(V + A + Z) where A is antheraxanthin, Z is zeaxanthin, and V is violaxanthin [38].

## Results

### Growth and recovery dynamics

**Fig 1:**
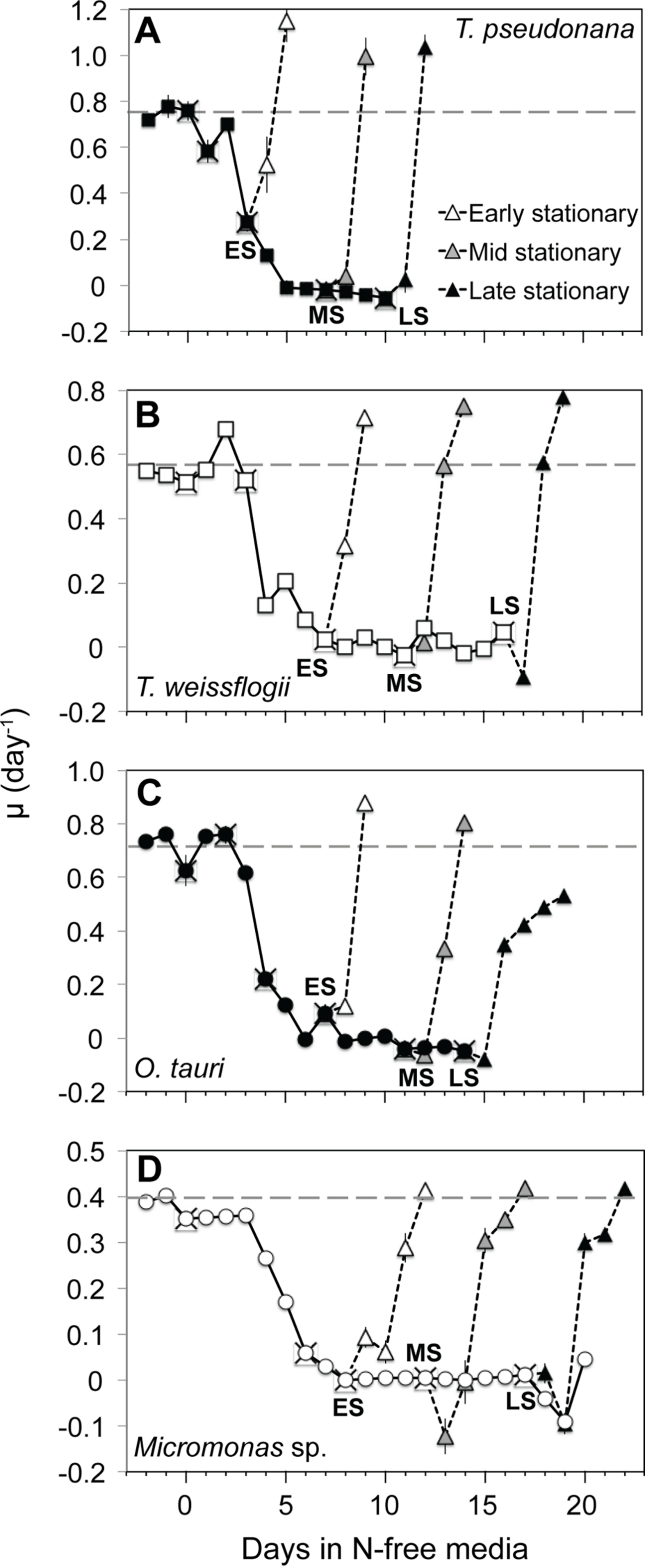
Growth rate (*μ*) from N-replete balanced growth to N starvation and in N-starved sub-cultures following the addition of nitrate in (A) *T*. *pseudonana*, (B) *T*. *weissflogii*, (C) *O*. *tauri*, and (D) *Micromonas* sp. (X) symbols indicate sampling points for cell composition and photochemistry. Recovery after the resupply of N is shown for subcultures collected at early (ES, white triangles), mid (MS, gray triangles), and late stationary (LS, black triangles) phases. The dashed line indicates the *μ*_*max*_ for a species determined during N-replete, balanced growth. Error bars indicate one standard deviation among triplicate cultures.

After nutrient-replete balanced growth (maximum growth rates shown in Table 3) and dilution with N-free media, all species ceased growth by the early stationary sampling point except *T*. *pseudonana*, where growth rate had declined by 83% and ceased the following day (Fig 1). Early stationary phase in all cultures was characterized by consistent minima in cellular N content (S1 Fig) and undetectable dissolved inorganic nitrogen (S1 Fig) while replete levels of dissolved orthophosphate (12.6 μM ± 3.7) and silica (10.8 μM ± 2.3) persisted throughout all experiments, indicating N starvation. Sub-samples of all cultures were resupplied with nitrate after each stationary phase sampling to observe growth and photochemical responses during recovery. The effect of progressive N starvation on recovery was compared on the basis of the recovery time (*T*_*R*_, d) to re-achieve *μ*_max_ (d^-1^) after resupply of N. Furthermore, we constructed a dimensionless recovery competence score (*RS* in Table 3, calculation shown in Table 2), to facilitate comparisons of recovery time across taxa. The recovery competence score (*RS*) is normalized to maximum growth rate, thus the percent change in this value indicates the change in a species rate of recovery with progressive N starvation that is comparable across species with different inherent growth and metabolic rates. Following resupply of N both diatom species recovered rapidly from all phases of N starvation, returning to their maximum growth rates within 2 days (Fig 1A and 1B) and showing only a modest decline in recovery competence (*RS*) with progressive N starvation (Table 3). *Micromonas* sp. also appeared resistant to progressive N starvation with similarly modest declines in *RS*, although it’s absolute recovery time was slower (~4–5 days to resume maximum growth) after all stationary phases (Fig 1D). In contrast, *O*. *tauri* showed a robust recovery from the early stationary phase of N-starvation by resuming maximum growth within 2 days with an *RS* of 0.77, but it’s recovery competence declined sharply with continued N-starvation such that at late stationary it failed to resume maximum growth even after 5 days of recovery.

**Table 3:** Growth rates and recovery scores.

Species, Growth Stage	*μ*_max_ (day^-1^)	*RS*	% Change in *RS*
***Thalassiosira pseudonana* (CCMP 1335)**	0.75 ± 0.03		-19.35 ± 7.22
Early Stationary		0.93 ± 0.06	
Mid Stationary		0.74 ± 0.04	
Late Stationary		0.75 ± 0.04	
***Thalassiosira weissflogii* (CCMP 1010)**	0.57 ± 0.06		-17.97 ± 9.23
Early Stationary		1.07 ± 0.16	
Mid Stationary		0.87 ± 0.13	
Late Stationary		0.88 ± 0.13	
***Ostreococcus tauri* (OTH 95)**	0.72 ± 0.06		-77.52 ± 12.07
Early Stationary		0.77 ± 0.09	
Mid Stationary		0.49 ± 0.06	
Late Stationary		0.17 ± 0.02	
***Micromonas* sp. (CCMP 2099)**	0.40 ± 0.01		-18.66 ± 4.80
Early Stationary		0.71 ± 0.03	
Mid Stationary		0.6 ± 0.02	
Late Stationary	** **	0.57 ± 0.02	

N-replete growth rate and recovery competence scores (*RS*) of after addition of nitrate to sub-cultures from each N-starved stationary growth phase. The derivation of the recovery competence score *RS* is shown in Table 2 and this score provides an index of the recovery from N starvation independent of the constitutive growth rate of each species.

### PSII Photochemistry

**Fig 2:**
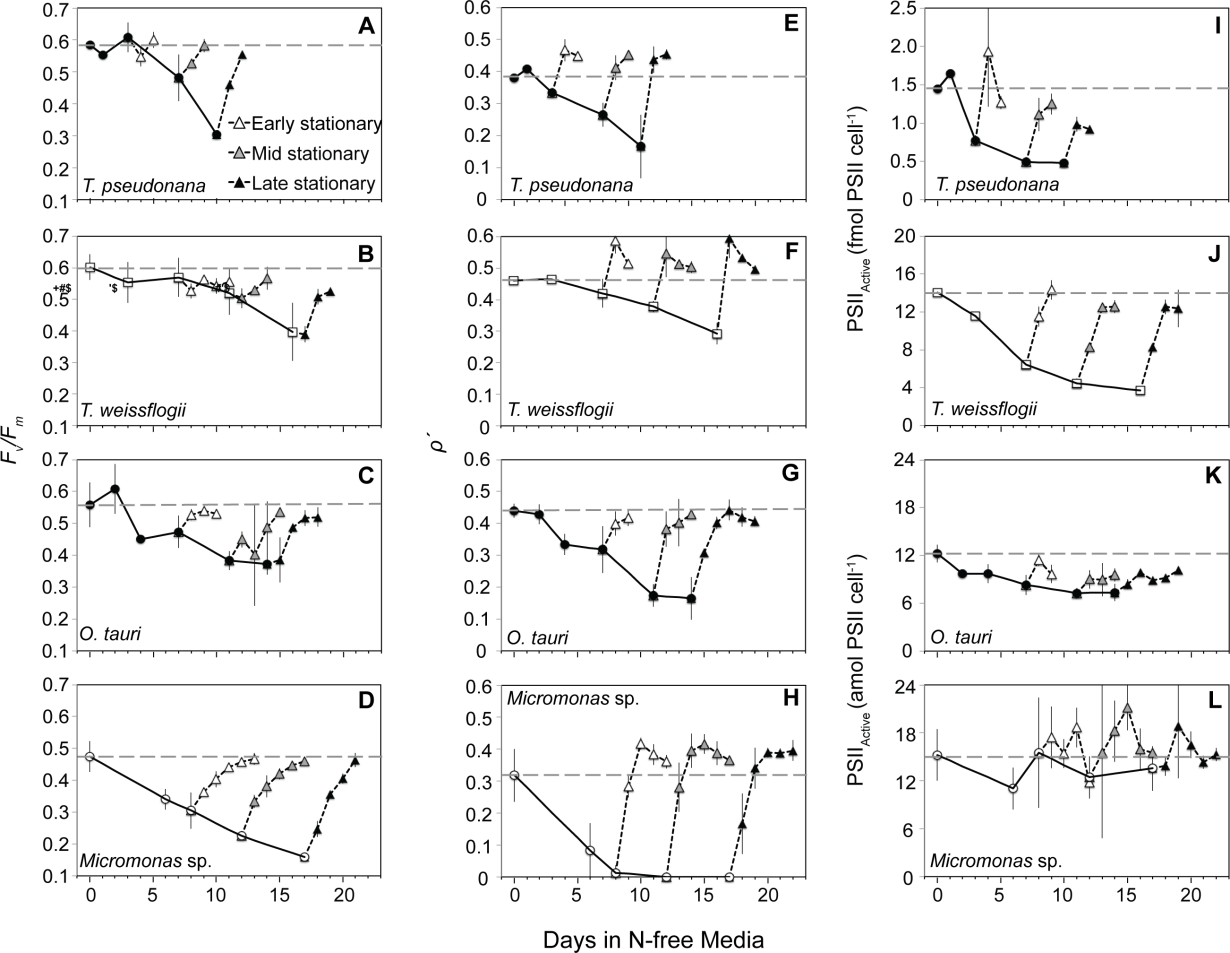
Changes in (A,B,C,D) the maximum quantum yield of the pool of PSII reaction centers (*F*_v_*/F*_m_), (E,F,G,H) the excitonic connectivity among PSII reaction centers (*ρʹ*), and (I,J,K,L) active PSII reaction center (PSII_Active_) content with N starvation and following the resupply of N. Symbols are the same as in Fig 1. The values shown for *ρʹ* at each sampling point were determined under low actinic light (8 and 21 *μ*mol photons m^-2^ s^-1^ for prasinophytes and diatoms respectively) as explained in the text. Error bars represent propagated standard error based on the calculated error of curve fitting by the FRRf software and the standard error among triplicate cultures.

The maximum quantum yield of the pool of PSII reaction centers (as *F*_v_*/F*_m_) declined in all species with N depletion (Fig 2A–2D), indicating the progressive loss, inactivation, or sustained quenching of PSII reaction centers. The excitonic connectivity of PSII reaction centers (*ρʹ*) declined with a similar pattern as *F*_v_*/F*_m_ for each species (Fig 2E–2H), likely due to the increasing distance among remaining active PSII [59]. *Micromonas* sp. displayed a lower N-replete *F*_v_*/F*_m_ and a roughly two-fold greater decline in *F*_v_*/F*_m_ with N starvation than the other species (Fig 2D). Additionally, *ρʹ* declined much more rapidly in *Micromonas* sp. and was 0 throughout stationary phase (Fig 2H). *Micromonas* sp. also differed from the other species as its cellular quota of active PSII reaction centers (PSII_Active_) showed no clear changes with N starvation, while PSII_Active_ declined in both diatom species and to a lesser extent in *O*. *tauri*.

After the resupply of N, *F*_v_*/F*_m_ recovered more slowly than growth in all species (Fig 1). Recovery of *ρʹ* was rapid and faster than the recovery of growth in all species, particularly in *Micromonas* sp. Recovery of PSII_Active_ was also more rapid than the recovery of growth in both diatom species, but considerably slower in *O*. *tauri*. PSII_Active_ did not return to near N-replete levels after mid or late stationary in *O*. *tauri* or after late stationary phase in *T*. *pseudonana*.

**Fig 3:**
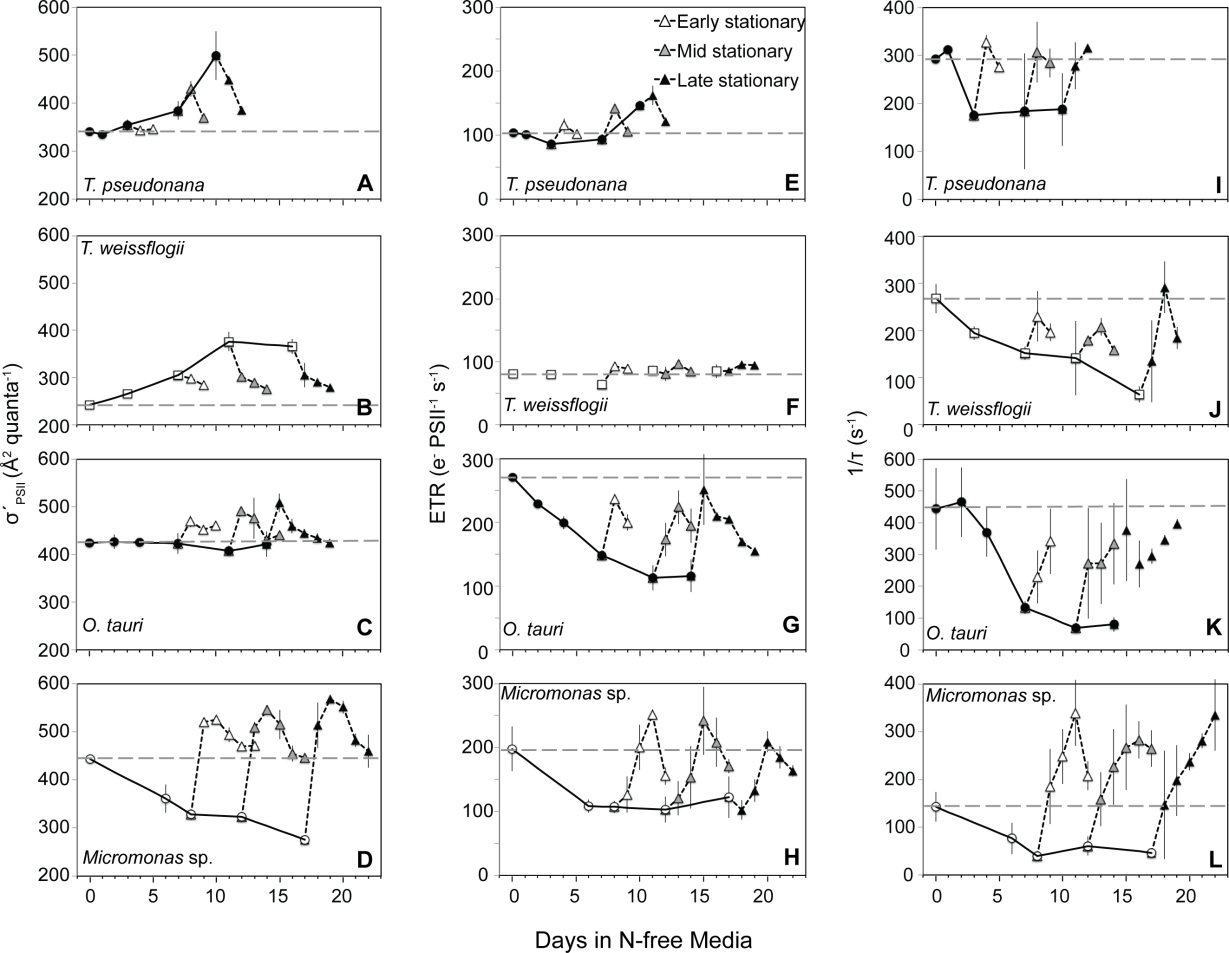
Changes in (A,B,C,D) the effective absorption cross-section of PSII photochemistry (*σʹ*_PSII_), (E,F,G,H) electron transfer rate from PSII (ETR), and (I,J,K,L) the rate constant for the reopening of PSII reaction centers (1/*τ*) with N starvation and during recovery following the resupply of N. Symbols for recovery response are the same as in Fig 1. The values shown for *σʹ*_PSII_ at each sampling point were determined under low actinic light (8 and 21 *μ*mol photons m^-2^ s^-1^ for prasinophytes and diatoms respectively) as explained in the text. Error bars represent propagated standard error based on the calculated error of curve fitting by the FRRf software and the standard error among triplicate cultures.

There were three distinct patterns in *σʹ*_PSII_ with N starvation and recovery among the four species examined (Fig 3A–3D). The effective absorption cross-section of remaining PSII reaction centers (*σʹ*_PSII_) increased with N starvation in the diatoms (Fig 3A and 3B). The *σʹ*_PSII_ of *O*. *tauri* remained high and nearly constant from exponential growth to N-depleted late stationary phase (Fig 3C). *Micromonas* sp. displayed a third response with *σʹ*_PSII_ decreasing sharply from N-replete growth to N-starved conditions (Fig 3D). With the resupply of N to both diatom species, *σʹ*_PSII_ returned to near N-replete levels, albeit more slowly than the recovery of growth. In *O*. *tauri σʹ*_PSII_ increased slightly during the first two days of recovery then returned to the consistent level maintained through N-replete and N-starved conditions. In contrast, the resupply of N to *Micromonas* sp. caused *σʹ*_PSII_ to increase rapidly, returning to and exceeding N-replete levels in only one day. This recovery response of *σʹ*_PSII_ in *Micromonas* sp. was much more rapid than the recovery response of growth or *F*_v_*/F*_m_ (Fig 3D).

Each species differed in the rates of electron transfer (ETR, Fig 3E–3H) from PSII and reopening kinetics (1/*τ*, Fig 3I–3L) of PSII during N starvation and recovery. The smallest changes in ETR with N stress were observed in diatoms as ETR increased slightly with progressive N starvation in *T*. *pseudonana* (Fig 3E) and did not change in *T*. *weissflogii* (Fig 3F). This smaller decline in ETR in diatoms upon N starvation was due to a decline in 1/*τ* (Fig 3I and 3J) and accordingly in the fraction of open PSII (*q*_p_) that was partially offset by an increased *σʹ*_PSII_ (Fig 3A and 3B) for the remaining open PSII reaction centers. In contrast, both prasinophytes species had large declines in ETR with N starvation (Fig 3G and 3H) driven by a decline in 1/*τ* (and accordingly in q_P_). After the resupply of N, *O*. *tauri* was the only species that did not show a rapid recovery in ETR and 1/*τ* to N-replete levels (Fig 3E–3L) with neither parameter returning to replete levels after any stage of N-starvation (Fig 3G and 3K).

### Photosynthetic pigments and light absorption

**Fig 4:**
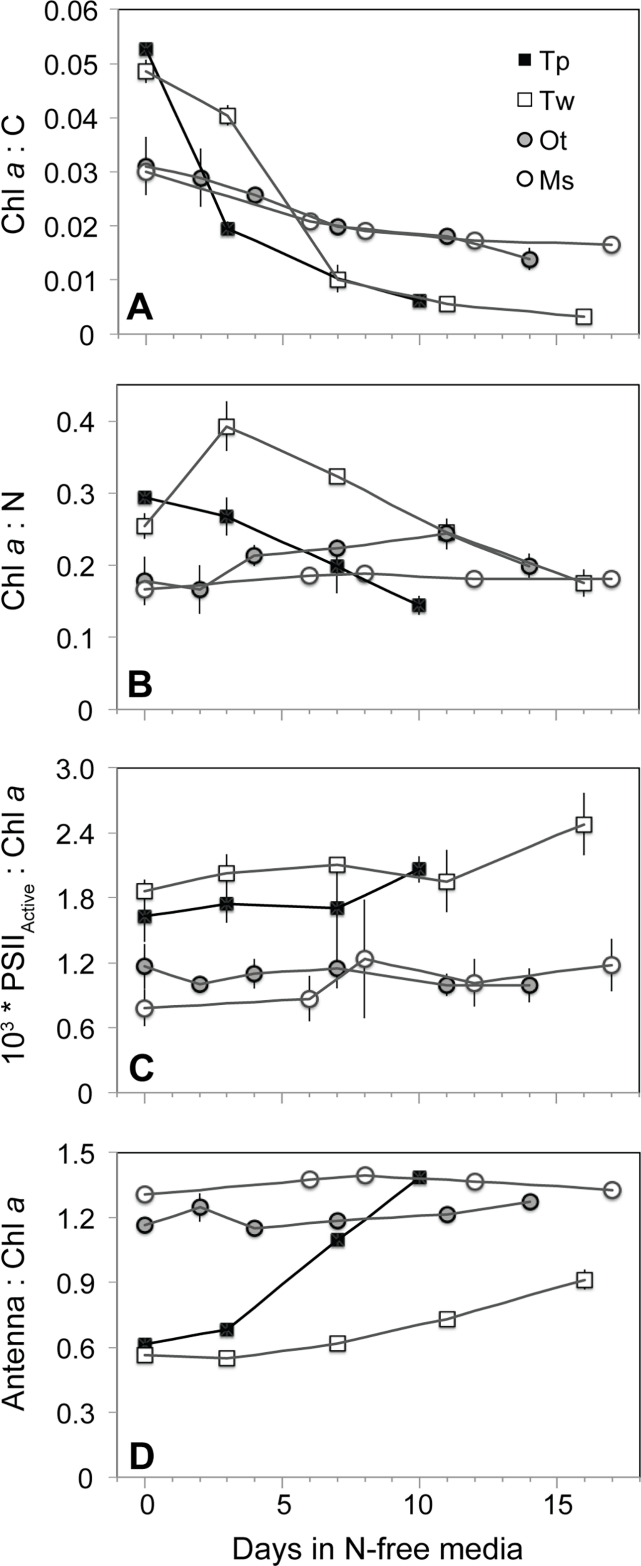
The change from N-replete growth to N starvation in molar ratios of (A) chl *a* content to cellular carbon, (B) chl *a* content to cellular nitrogen, (C) the cellular content of active PSII reaction centers to chl *a*, and (D) pigment content associated with light harvesting antenna complexes to chl *a*. Error bars indicate one standard deviation among triplicate cultures.

Pigment composition and its variation with N starvation were similar between diatom species and between prasinophyte species, but distinct between these two classes. Diatoms had a higher mean N-replete carbon-normalized chlorophyll *a* (Chl *a*) content (Chl *a*:C, mass:mass) of 0.051 ± 0.001 compared to prasinophytes (0.031 ± 0.002). The subsequent decline in Chl *a*:C was also much larger in the diatoms (Fig 4A). Despite an initial increase in nitrogen-normalized Chl *a* content (Chl *a*:N, mass:mass) in *T*. *weissflogii*, this value also decreased with N starvation in both diatoms, indicating a more rapid decline in Chl *a* than in other components of cellular N (Fig 4B). In contrast, Chl *a*:N increased slightly in *O*. *tauri* and was invariant in *Micromonas* sp. with N-starvation. Similar patterns for each species were also observed in carbon- and nitrogen-normalized total pigment content (not shown). The larger decline in Chl *a*:C in the diatoms was due primarily to the greater mean decrease in diatom cellular Chl *a* content (-77.1 ± 1.8%) and relatively small change in prasinophyte cellular Chl *a* content (-35.3 ± 5.9%) rather than differences in carbon accumulation (not shown). The proportion of remaining active PSII reaction centers (PSII_Active_:Chl *a*) increased in diatoms yet showed little to no change in prasinophytes (Fig 4C).

The amount of light harvesting antenna relative to PSII reaction centers in each species was estimated as the molar ratio of pigments primarily associated with light harvesting complexes to Chl *a* (Antenna:Chl *a*). Both prasinophytes exhibit a comparatively larger light harvesting antenna (>Antenna pigment:Chl *a*) than the diatoms during N-replete growth (Fig 4D). Among the prasinophytes, N-replete Antenna pigment:Chl *a* was consistently higher in *Micromonas* sp. (1.35 ± 0.01) compared to *O*. *tauri* (1.21 ± 0.01). With progressive N starvation, prasinophytes showed no change in Antenna pigment:Chl *a* while diatoms displayed a large increase in Antenna pigment:Chl *a* as their Chl *a* content (and likely PSII reaction centers) declined more than antenna-associated pigments.

A lower light absorption normalized to chl *a* (${\bar{a}}^{*}$) was observed in the diatoms compared to the prasinophytes under N-replete conditions and is consistent with a greater amount of pigment packaging likely to accompany their greater cell size (S2 Fig). Concomitant with chlorosis following N-deprivation, ${\bar{a}}^{*}$ increased all species.

### Induction of non-photochemical quenching and xanthophyll cycle

The quantum yield of energy-dependent non-photochemical quenching (NPQ) of fluorescence (***Φ***_NPQ_) was estimated throughout each N starvation experiment using FRRf parameters (See Methods and Table 2). The primary mechanism underlying ***Φ***_NPQ_ is the rapid, energy-dependent de-epoxidation of xanthophyll pigments [49], which was estimated as the de-epoxidation state (DES) of these pigments. Changes in the photoprotective pigment lutein (present in prasinophytes) were also examined in the context of NPQ as lutein has been implicated in slow acting sustained NPQ mechanisms [38,60,61].

**Fig 5:**
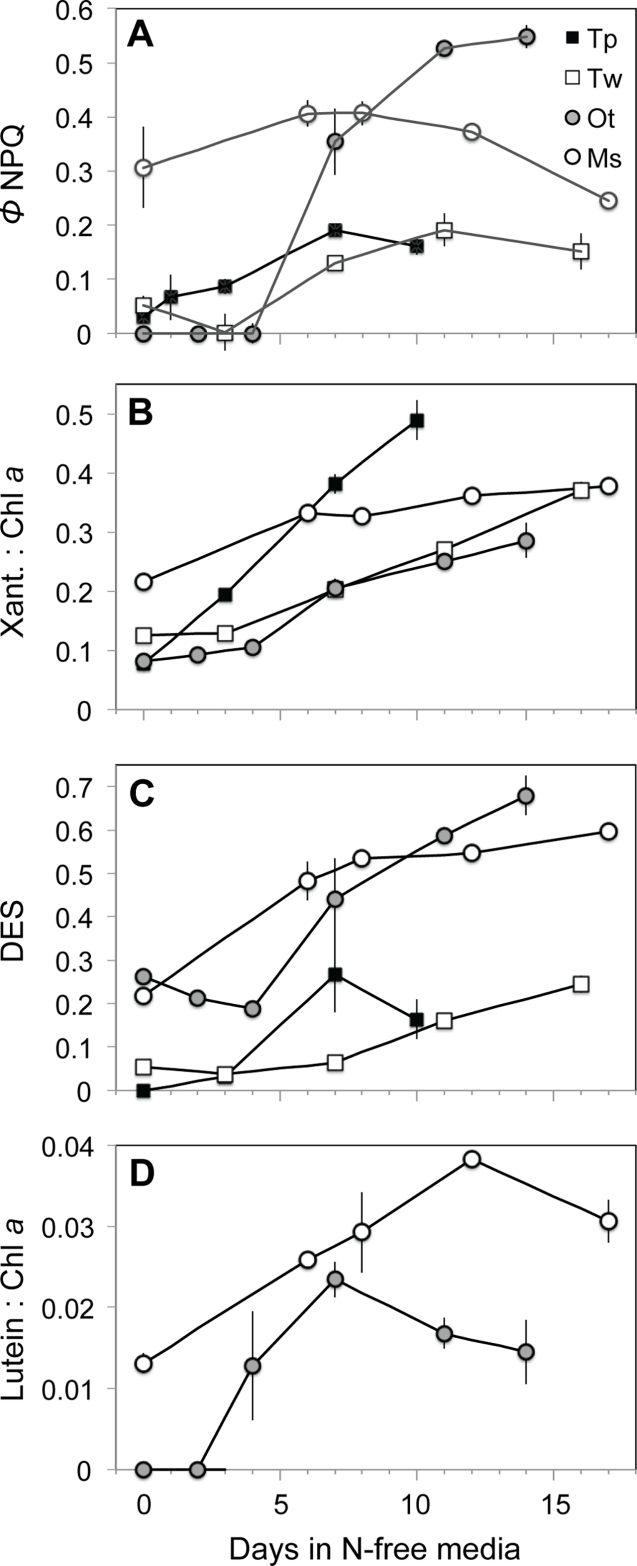
Non-photochemical quenching (NPQ) activity in N starvation experiments as shown by of (A) FRRf-based measurement of excitation energy allocation to energy-dependent NPQ (***Φ***_NPQ_), (B) the molar ratio of xanthophyll cycle pigments to chl *a*, (C) the de-epoxidation state (DES) of xanthophyll cycle pigments, and (D) the molar ratio of lutein to chl *a*. Error bars indicate standard deviation among triplicate cultures.

Three distinct patterns of NPQ emerged during N-starvation among the four species examined. The diatoms were similar in displaying relatively little energy allocation to ***Φ***_NPQ_ under N-replete growth and a modest increase with N starvation (Fig 5A). This reallocation appeared to be driven by increases in Xanth:Chl *a* as well as an increase in DES (Fig 5B and 5C). *O*. *tauri* displayed a similar but more dramatic pattern (Fig 5A–5C) with no measurable energy-dependent NPQ under N-replete growth and throughout the early stages of N depletion followed by a large reallocation of excitation energy to ***Φ***_NPQ_ in stationary phase as DES, Xanth:Chl *a*, and cellular xanthophyll content (not shown) increased. The distinct third type of response observed in *Micromonas* sp. included a much higher ***Φ***_NPQ_ and Xanth:Chl *a* under N-replete growth and little change in ***Φ***_NPQ_, Xanth:Chl *a* with the onset of N starvation.

Lutein:Chl *a* (Fig 5D) increased in both prasinophyte species from exponential to stationary growth phases. However, lutein content declined after early stationary phase in *O*. *tauri*, but continued to increase in *Micromonas* sp. until mid-stationary phase. The relative increase in lutein and thus lutein:Chl *a* was also consistently higher in *Micromonas* sp. than *O*. *tauri* with N starvation.

### Re-allocation of excitation energy during starvation and recovery

**Fig 6:**
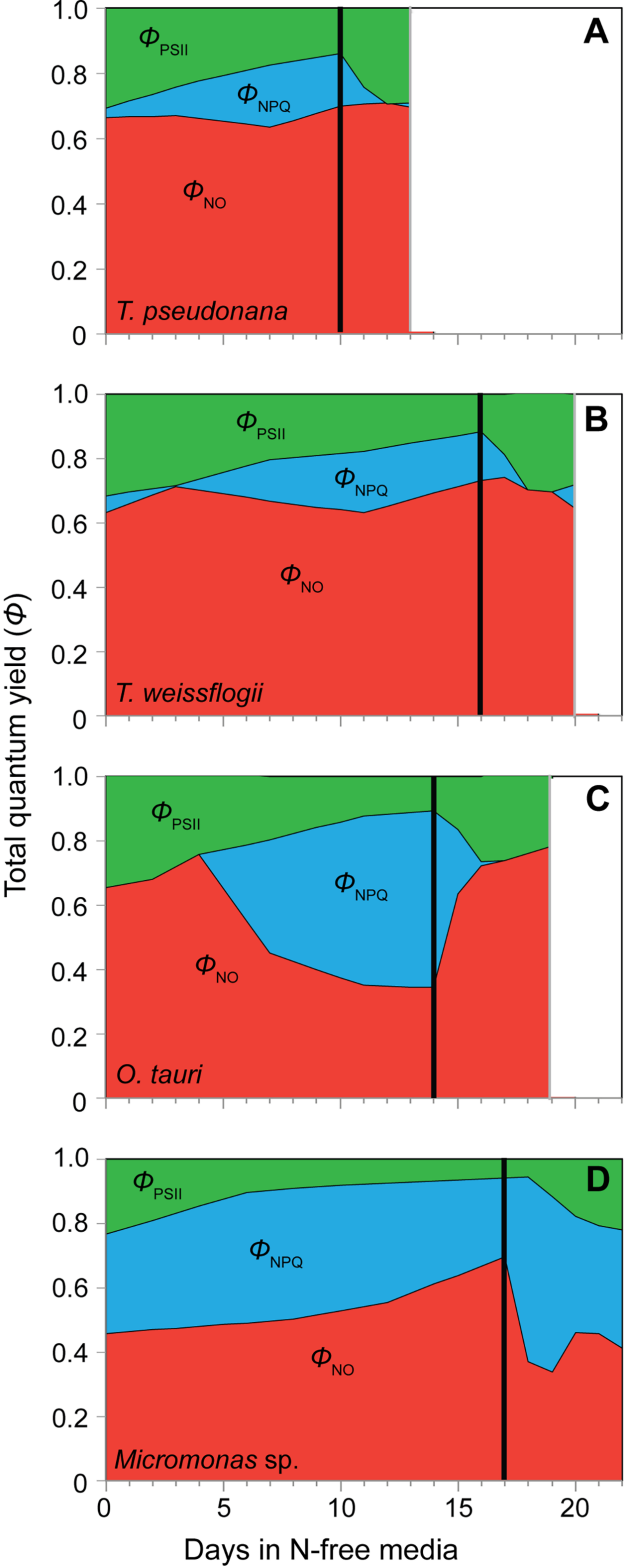
The relative distribution of excitation energy among PSII photochemistry (*Φ*_PSII_, green shading), dissipation as heat via energy-dependent non-photochemical quenching (*Φ*_NPQ_, blue shading), and dissipation as heat and fluorescence as constitutive, energy-independent non-photochemical quenching (*Φ*_NO_, red shading) in (A) *T*. *pseudonana*, (B) *T*. *weissflogii*, (C) *O*. *tauri*, and (D) *Micromonas* sp. with the onset of N-starvation and during recovery following the resupply of N. *Fs and Fm’* values used in to calculate these quantum yields were determined at growth irradiance (85 *μ*mol photons m^-2^ s^-1^). Only recovery from late stationary phase N starvation is shown for simplicity.

The various strategies of each species for allocating excitation energy during N starvation were derived from FRRf parameters [49,50] and is shown in Fig 6. Only the recovery from late stationary phase is shown for clarity, as recovery responses in energy allocation were similar across stationary phases. The diatom species exhibited similar patterns of reallocating excitation energy from PSII photochemistry (*Φ*_PSII_) to *Φ*_NPQ_ while constitutive non-regulated dissipation (*Φ*_NO_) showed little change and remained high (Fig 6A and 6B). The more dramatic induction of the xanthophyll cycle in *O*. *tauri* with N starvation is reflected in its large reallocation of energy to *Φ*_NPQ_, a slightly greater decline in *Φ*_PSII_ than seen in the diatoms, and a very large decline in *Φ*_NO_ (Fig 6C). The reallocation of excitation energy in the diatoms and *O*. *tauri* was rapidly reversed after resupply of N, further indicating that these species *Φ*_NPQ_ dynamics are due to the rapidly inducible xanthophyll cycle (Fig 6A–6C). As with its dynamics in PSII photochemistry, *Micromonas* sp. showed a distinct photophysiological strategy in its distribution of excitation energy. *Micromonas* sp. had a constitutively high capacity for *Φ*_NPQ_ under N-replete conditions (Fig 5A, Fig 6D). Additionally, *Micromonas* sp. showed a large increase in non-regulated, energy-independent dissipation (*Φ*_NO_) throughout N starvation as *Φ*_PSII_ and *Φ*_NPQ_ declined. This increase in *Φ*_NO_ appears to be part of an induced mechanism rather than progressive photoinhibition as it was quickly reversed after the resupply of N, just as the reallocation to *Φ*_NPQ_ due to xanthophyll cycle activity was quickly reversed in the other three species upon recovery (Fig 6D).

## Discussion

We examined photosynthetic responses to N starvation within two important phytoplankton classes from distinct phyla with differing photosynthetic structures. The phylogeny, cell size and a species’ adaptation to the nutrient regime of its niche are factors that could be expected to affect these photosynthetic responses. Among the diatoms studied, *T*. *pseudonana* is a small coastal species while *T*. *weissflogii* is a medium-sized open ocean species (Table 1). Among the prasinophytes, *O*. *tauri* is the smallest free-living eukaryote known from a shallow, eutrophic, coastal lagoon, while the larger *Micromonas* sp. dominates the picophytoplankton size class in the Arctic Ocean [42] (Table 1). Environmental niche and cell size differences within each class appear to have little effect on the N stress response of photosynthetic structures as diatoms displayed large changes in pigment composition during N starvation, while both prasinophytes were more homoeostatic. However, PSII photochemistry, photoprotection, and the recovery of growth and photosynthetic function from N starvation varied greatly at the species level, particularly between the prasinophytes. While structural variability affects how species within each phylum utilize and compete for N and other resources, our findings suggest that variation in photochemistry and photoprotection at the species or niche level determines the ability to endure and recover from N starvation.

The prasinophytes *O*. *tauri* and *Micromonas* sp., have a very different photosynthetic response to dynamic N stress compared to the diatoms in this study, other diatoms [11,13] and 9 other microalgal phyla and classes [12,16,62]. Both prasinophytes displayed little change in pigment content with N starvation. The prasinophytes also displayed a decoupling of light absorption from PSII activity consistent with their large diversions of excitation energy away from PSII photochemistry. The larger decline of active PSII reaction centers relative to light harvesting antenna in *O*. *tauri* would be expected to increase the effective absorption cross-section of each its remaining active PSII reaction centers (*σʹ*_PSII_), as observed for diatoms in this study (Fig 3A and 3B) and others [11,13] as well as in many other taxa [12,16,62]. Instead, *σʹ*_PSII_ is invariant in *O*. *tauri* likely due to its large diversion of excitation energy to NPQ via the xanthophyll cycle, effectively decoupling light absorption and PSII photochemistry. In *Micromonas* sp. both the antenna and the content of active PSII are maintained during N starvation, which would be expected to result in little change in *σʹ*_PSII_. Instead, we find a decline in *σʹ*_PSII_ for *Micromonas* sp. that can also be explained by its constitutively high xanthophyll cycle activity and its diversion of excitation energy to energy-independent dissipation (***Φ***_NO_).

In a previous study that observed similar decoupling of light absorption and PSII function in *O*. *tauri* and *Micromonas pusilla* (a temperate species) with steady-state N limitation [63], the authors noted that this response requires large energy dissipation mechanisms to prevent oxidative stress. Our findings demonstrate these photoprotective mechanisms, their subsequent diversions of excitation energy that explain the observed decoupling, and their relative effectiveness during nutrient stress. In N-starved *O*. *tauri*, there is a large induction of xanthophyll cycle activity (Fig 5B and 5C) and a reallocation of excitation energy to energy-dependent NPQ (***Φ***_NPQ_, Fig 5A, Fig 6C) that relaxes when N is resupplied. By late stationary phase, *O*. *tauri* displayed poor recovery of growth (Fig 1C) and diminished ETR (Fig 3H and 3K) after N was resupplied, indicating that its ***Φ***_NPQ_-based response fails to provide sustained protection to photosynthetic components down-stream of PSII. Unlike *O*. *tauri*, *Micromonas* sp. maintains a high ***Φ***_NPQ_ through xanthophyll cycle activity during N-replete growth and makes a comparatively small reallocation of excitation energy to this mechanism with no increase in xanthophyll content at the onset of N starvation (Fig 5A and 5B). This strain of *Micromonas* sp. also displays high xanthophyll cycle activity during nutrient-replete growth at the same temperature and much lower (30 *μ*mol photons m^-2^ s^-1^) growth irradiance [64], indicating that this is a constitutive feature of its photophysiology. Prolonged N starvation in *Micromonas* sp. induces an additional and sustained form of photoprotection that reallocates a large portion of excitation energy to energy-independent NPQ (***Φ***_NO_, Fig 6D) and relaxes rapidly with N resupply. This additional form of energy dissipation may help *Micromonas* sp. to recover growth and photosynthetic function after prolonged N-stress with little alteration of its photosynthetic pigment-protein complexes.

We propose that the induction of energy-independent NPQ in *Micromonas* sp. for photoprotection represents a sustained form of photoprotection as observed in vascular plants like over-wintering evergreens and avocado [65–67] as well as an Antarctic strain of the green microalga *Chlamydomonas* [68]. These mechanisms provide a “locked-in” [69] form of energy dissipation by NPQ as a general strategy for coping with prolonged stress [70]. This sustained photoprotection is thought to involve the accumulation of lutein [60,69]) or zeaxanthin [65, 71,72] and structural changes in the light harvesting antenna complexes adjacent to PSII reaction centers that allow these pigments to directly interact with and dissipate energy from PSII [61,73,74]. In this study *Micromonas* sp. displayed large increases in lutein and DES (conversion of other xanthophylls to zeaxanthin) and maintained a high lutein:Chl *a* throughout N starvation. A sustained quenching mechanism at PSII in *Micromonas* sp. would also explain the loss of excitonic connectivity among PSII (Fig 2H), the large reallocation to energy-independent thermal losses (***Φ***_NO_, Fig 6D) and the decrease in *σʹ*_PSII_ (Fig 3D) despite maintaining light harvesting pigment capacity (Fig 4D). Additionally, *ρʹ*, *σʹ*_PSII_, and ***Φ***_NO_ rapidly returned to N-replete levels much faster than did growth rate or *F*_v_*/F*_m_, indicating that their recovery is not due to replacement of photodamaged PSII or growth, but rather to the relaxation of an induced quenching mechanism.

Diatoms present a very different strategy than prasinophytes for allocating excitation energy in order to cope with N stress. Both diatoms allocated much less excitation energy to ***Φ***_NPQ_ via xanthophyll cycle activity (Figs 5A and 6) during N-replete growth as compared to *Micromonas* sp. The diatom's subsequent induction of this energy-dependent form of NPQ was also modest compared to the large up-regulation in *O*. *tauri*. Diatoms also exhibit far greater declines in light harvesting complexes and PSII reaction centers during N starvation than prasinophytes as indicated by pigment composition, lowering their overall light absorption and excitation energy. Diatoms also appear to rely on large constitutive losses of excitation energy as shown by their consistently high ***Φ***_NO_. These large changes in photosynthetic structure and high ***Φ***_NO_ are both likely a result of greater cell size and pigment packaging, providing diatoms with a larger photosynthetic apparatus and greater energetic inefficiencies through thermal losses. This same advantage of cell size provides decreasing Chl *a*-specific light absorption [26] and lower susceptibility to photoinactivation of PSII with increasing cell size [27]. The combination of constitutive resistance to photosynthetic damage and effective, inducible photoprotective mechanisms seem to confer a robust ability to resist and recover from N starvation on diatoms.

The distinct photoprotective mechanisms observed at the species-level in this study may reflect niche adaptation. Rapidly induced, energy-dependent photoprotection through the xanthophyll cycle may be advantageous in the shallow well-mixed habitat of *O*. *tauri*, where a cell can experience rapid changes (seconds to minutes) in irradiance [1,32]. Reliance on large increases in xanthophyll cycle NPQ has also been observed in *Ostreococcus* in response to excess irradiance [38,75]. In coastal diatoms, a large and more dynamic capacity for xanthophyll cycle NPQ has been linked to their ability to cope with variable light stress better than open ocean diatoms [32]. The photoprotective strategy of *Micromonas* sp. may reflect adaptation to the cold, low irradiance waters from which it was isolated [42]. A sustained quenching mechanism may be uniquely suited to this low light oligotrophic habitat, where there is less risk of a sudden increase in light and a large light harvesting antenna may be safely maintained during N starvation. Similar sustained quenching mechanisms have been observed in an Antarctic strain of the chlorophyte *Chlamydomonas* [68,76], and in several species of over-wintering plants [66,68,70] indicating that it may also be an adaptation for sustaining photosynthetic systems at very low temperatures.

The overall photosynthetic responses to N-starvation for *T*. *pseudonana*, *T*. *weissflogii*, *O*. *tauri*, and *Micromonas* sp. are informative in the contexts of cellular N allocation and resource competition. The response to N starvation in diatoms is similar to other microalgal groups examined to date in that it involves a greater reallocation of cellular N from photosynthesis [12,14] (Fig 4B). This strategy allows more N from photosynthetic components to be reutilized for N acquisition mechanisms [14], thus increasing N affinity in response to external depletion. The constraints of cell size that compel diatoms to reallocate large portions of photosynthetic resources during N starvation may also result in larger opportunity costs upon recovery by requiring cells to rebuild more photosynthetic structures. However, the N-starved diatoms examined in this study were able to rapidly resume maximum growth upon the resupply of N (Fig 1) prior to all components of photosynthetic function returning to N-replete levels (Figs 2 and 3), indicating an ability to mitigate these higher opportunity costs or not require full photosynthetic recovery for the recovery of growth. A similar hysteresis between growth and PSII function upon recovery from N starvation was observed in a green alga with a similar N starvation response [14]. This may indicate that resistance to N starvation or greater investment upon recovery in cell components other than photosynthetic structures can mitigate the high material opportunity costs of large changes in photosynthetic structures.

A large reallocation of cellular N to N uptake mechanisms may be of less value to prasinophytes, which already possess relatively high nutrient uptake affinity attributable to their small size and high surface:volume ratio [77]. Instead, prasinophytes may maintain photosynthetic structures to remain primed for recovery [63], rather than relying upon reallocation of photosynthetic N to resist starvation. By maintaining photosynthetic structures during N starvation, prasinophytes avoid the opportunity costs, in terms of available N, energy and time, of rebuilding these structures. The poor recovery of growth, ETR, and reopening of PSII in *O*. *tauri* at N-starved late stationary phase may be evidence that this priming strategy coupled with a reliance on energy-dependent, ***Φ***_NPQ_-based photoprotection is insufficient for coping with prolonged nutrient stress. By contrast, the sustained photoprotective quenching mechanism of *Micromonas* sp. may allow it to maintain a relatively large light harvesting apparatus while also being both primed for recovery and adequately protected from photodamage. Polar phytoplankton like *Micromonas* sp. have also been shown to have very high cellular protein content (and thus high N content) to overcome temperature limitation of rate processes and biosynthesis [76,78]. In particular, the polar *Micromonas* sp. used in this study has been shown to maintain a large pool of inactive PSII reaction center protein (D1) to compensate for its slow D1 synthesis and repair rates [64]. Thus the photosynthetic response of *Micromonas* sp. to N starvation appears part of an overall strategy of reducing opportunity costs and metabolic turnover of N pools while providing sufficient photoprotection to ensure survival and recovery.

## Conclusions

We demonstrate that the photosynthetic response to dynamic nutrient conditions varies in complex ways with respect to taxa and niche and may be a key feature determining the success of a phytoplankton species. Both a coastal and open ocean diatom displayed large declines in photosynthetic pigments while both a coastal and Arctic open ocean prasinophyte showed similarly small changes in photosynthetic pigments. This similarity in pigment dynamics within diatoms and prasinophytes indicates the importance of cell size and phylogeny in determining changes in the material structure of photosynthesis during N-stress. These changes in photosynthetic structures also reveal a potential link between photosynthetic and N metabolism strategies. Larger reductions in photosynthetic structures in diatoms may allow greater reallocation of cellular N and lowering of N demand during starvation, while the more invariant strategy of prasinophytes may reduce the opportunity costs associated with rebuilding photosynthetic structures upon recovery. Despite these similarities within phyla, successful survival and recovery from N-starvation seems to rely more heavily on the interaction between environmental conditions and photoprotective strategies at the species level, particularly among prasinophytes. The prasinophyte from a more variable and nutrient rich environment, *O*. *tauri*, opted for a short-term photoprotective strategy and showed a large decline in its ability to recover from prolonged N-starvation. The prasinophyte from a more stable, oligotrophic system, *Micromonas* sp., employed a strategy of long-term photoprotection through sustained NPQ allowing it to maintain much of its N-rich photosynthetic apparatus as well as its ability to recover from N-starvation. We also show that *Micromonas* sp., the most abundant picophytoplankter in the Arctic ocean, utilizes a photosynthetic mechanisms rarely observed in phytoplankton, highlighting the need for more examination of the physiological strategies that allow prasinophytes to dominate many picophytoplankton communities.

## Supporting information

S1 Fig Decline in cellular and external nitrogen. The decline in (A) the ratio of cellular nitrogen quota at a sampling point (Q_N_) to the maximum nitrogen quota measured during N-replete, balanced exponential growth (Q_N_ Max) and (B) media concentration of dissolved inorganic nitrogen (DIN) which includes nitrate, nitrite, and ammonium. Q_N_: Q_N_ max is shown rather than Q_N_ so that all species can be viewed on the same scale. Error bars indicate one standard deviation among triplicate cultures.
(TIFF)

S2 FigChange in light absorption cross-section.The change in the spectrally averaged Chl *a*-specific light absorption cross section (${\bar{a}}^{*}$) in N-starved batch cultures of *T*. *pseudonana* (Tp), *T*. *weissflogii* (Tw), *O*. *tauri* (Ot), and *Micromonas* sp. (Ms). Error bars indicate one standard deviation.(TIFF)
