# Correction: Nitrogen starvation induces distinct photosynthetic responses and recovery dynamics in diatoms and prasinophytes

**DOI:** 10.1371/journal.pone.0224489

**Published:** 2019-10-25

**Authors:** Justin D. Liefer, Aneri Garg, Douglas A. Campbell, Andrew J. Irwin, Zoe V. Finkel

In Fig 2, incorrect units are shown for panels I, J, K, and L. The units for the vertical axis for panels I and J should be: amol PSII cell^-1^. The units for the vertical axis for panels K and L should be: zmol PSII cell^-1^. Please see the correct Fig 2 here.

**Fig 2:**
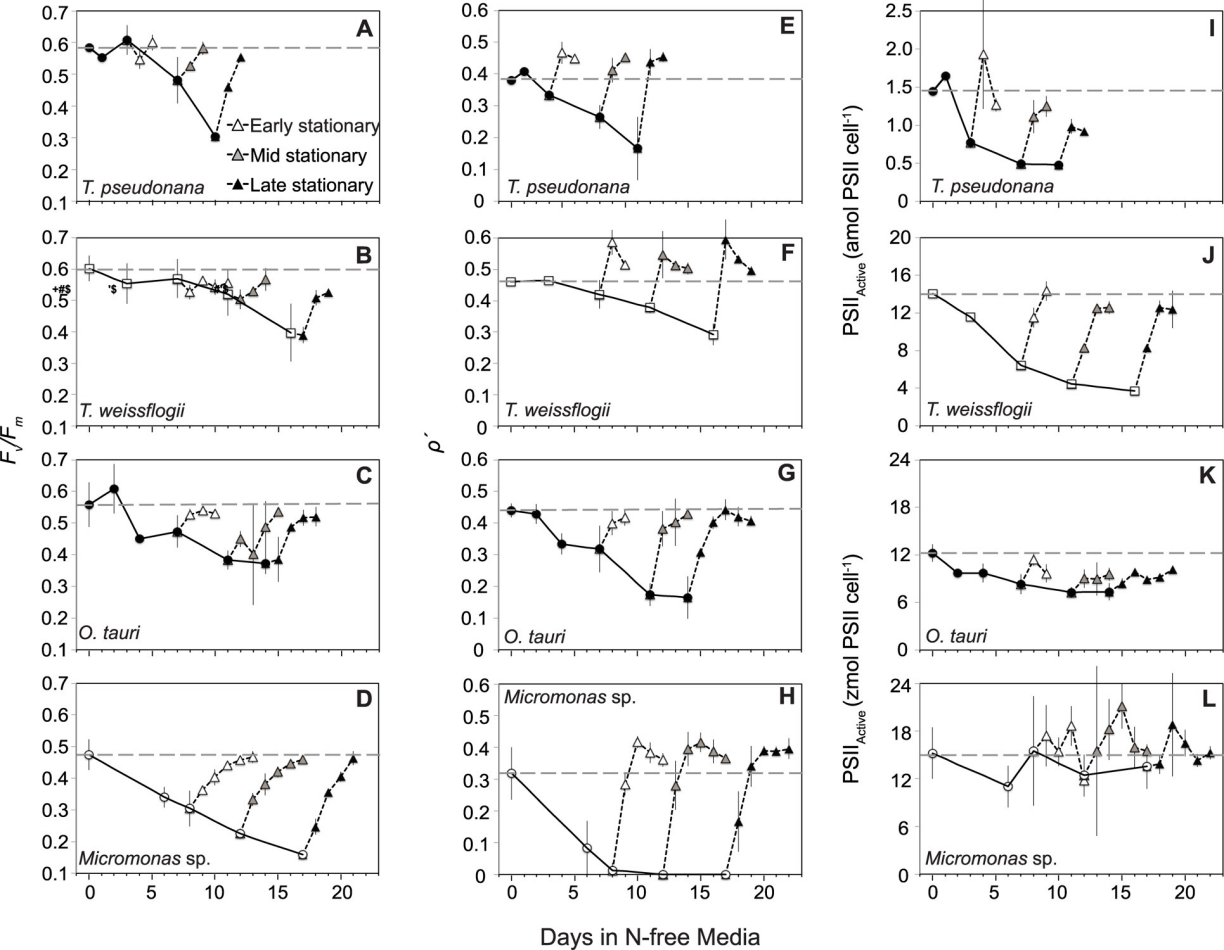
Changes in (A,B,C,D) the maximum quantum yield of the pool of PSII reaction centers (*F*_v_*/F*_m_), (E,F,G,H) the excitonic connectivity among PSII reaction centers (*ρ*′), and (I,J,K,L) active PSII reaction center (PSII_Active_) content with N starvation and following the resupply of N. Symbols are the same as in Fig 1. The values shown for *ρ*′ at each sampling point were determined under low actinic light (8 and 21 *μ*mol photons m^-2^ s^-1^ for prasinophytes and diatoms respectively) as explained in the text. Error bars represent propagated standard error based on the calculated error of curve fitting by the FRRf software and the standard error among triplicate cultures.

## References

[1] Liefer JD, Garg A, Campbell DA, Irwin AJ, Finkel ZV (2018) Nitrogen starvation induces distinct photosynthetic responses and recovery dynamics in diatoms and prasinophytes. PLoS ONE 13(4): e0195705 10.1371/journal.pone.0195705 29641594 PMC5895044

