# Supplementary figures and images for: Nitrogen starvation induces distinct photosynthetic responses and recovery dynamics in diatoms and prasinophytes
Source: PLoS One. 2018 Apr 11;13(4):e0195705. doi: 10.1371/journal.pone.0195705 (PMC5895044; doi:10.1371/journal.pone.0195705)

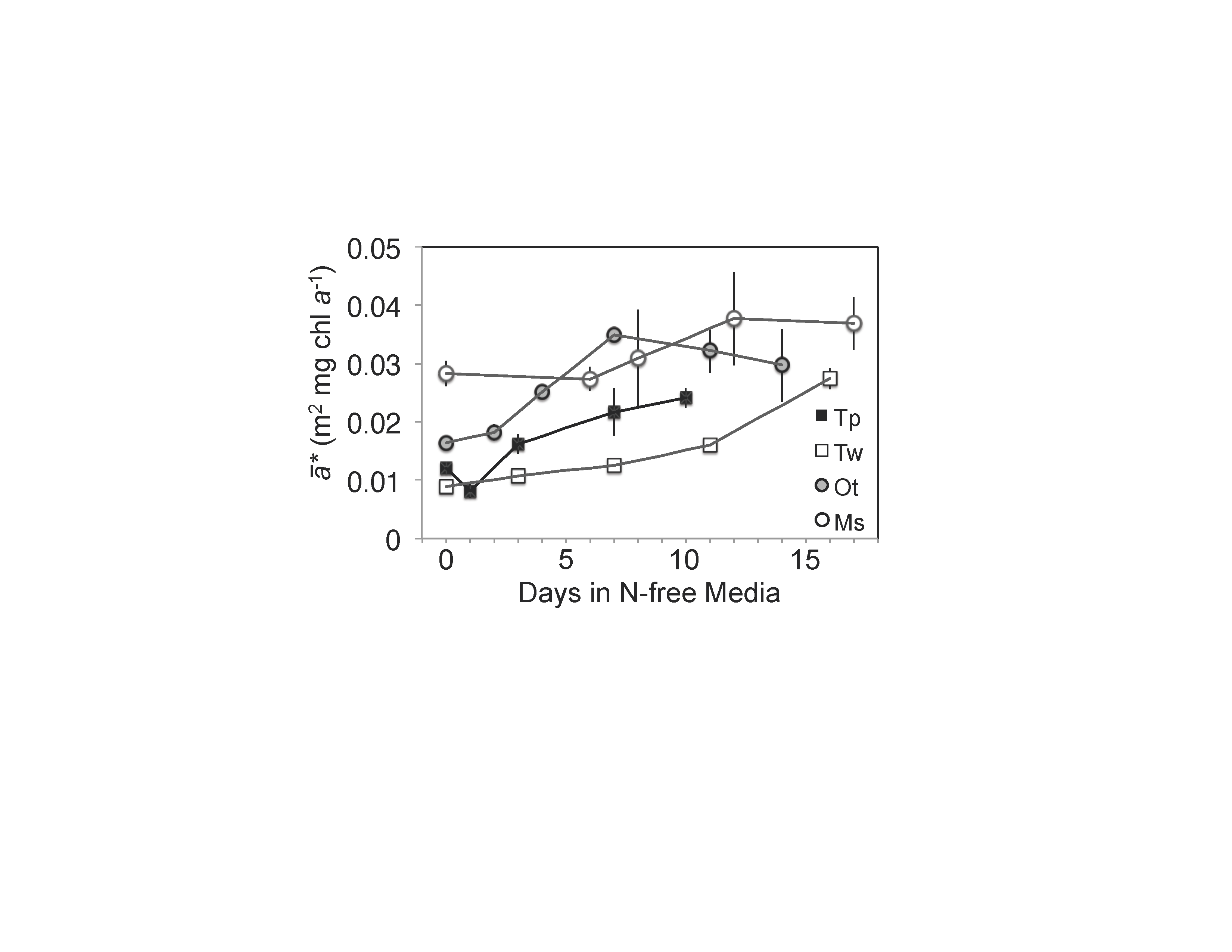

Supplement: S1 Fig — The decline in (A) the ratio of cellular nitrogen quota at a sampling point (QN) to the maximum nitrogen quota measured during N-replete, balanced exponential growth (QN Max) and (B) media concentration of dissolved inorganic nitrogen (DIN) which includes nitrate, nitrite, and ammonium. QN: QN max is shown rather than QN so that all species can be viewed on the same scale. Error bars indicate one standard deviation among triplicate cultures. (TIFF) [file pone.0195705.s001.tiff]

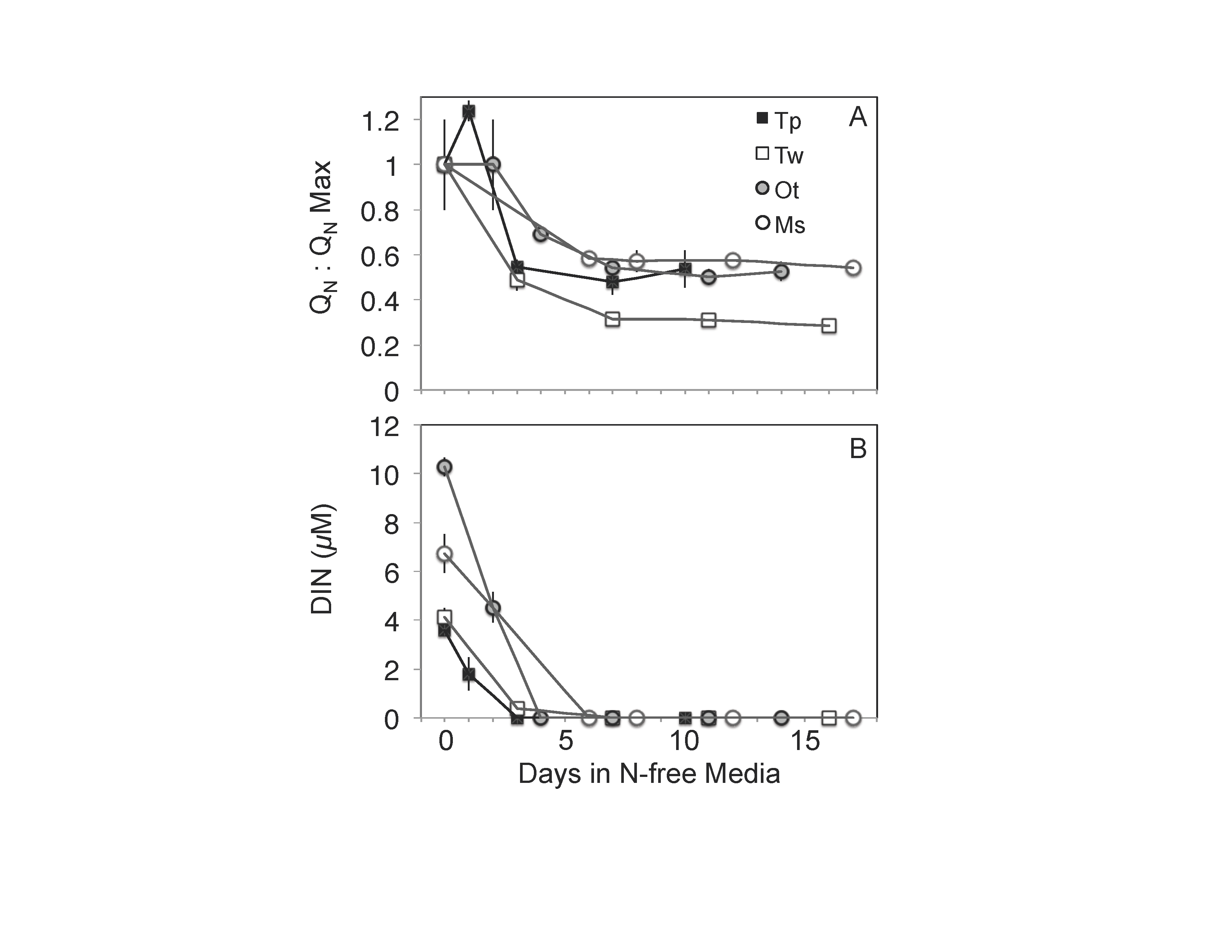

Supplement: S2 Fig — The change in the spectrally averaged Chl a-specific light absorption cross section (a¯*) in N-starved batch cultures of T. pseudonana (Tp), T. weissflogii (Tw), O. tauri (Ot), and Micromonas sp. (Ms). Error bars indicate one standard deviation. (TIFF) [file pone.0195705.s002.tiff]
